# A systematic review of the effects of artemether-lumefantrine on gametocyte carriage and disease transmission

**DOI:** 10.1186/1475-2875-13-S1-P59

**Published:** 2014-09-22

**Authors:** Michael Makanga

**Affiliations:** 1 European & Developing Countries Clinical Trials Partnership, Cape Town, South Africa

## Background

Despite significant advances made in the prevention and treatment of malaria in recent years, these successes continue to fall short of the World Health Organization (WHO) goals for malaria control and elimination. For elimination strategies to be effective, limited disease transmission, achieved through rapid reduction in infectious parasite reservoir and decreased gametocyte carriage, will be critical. Artemisinin-based combination therapy (ACT) forms the cornerstone of WHO-recommended treatment for uncomplicated *Plasmodium falciparum* malaria, and in combination with other effective interventions will undoubtedly play a vital role in elimination programmes. The gametocytocidal properties of ACTs are a bonus attribute; there is epidemiological evidence of reductions in malaria incidence and transmission in African regions since the introduction of these agents. Many studies and analyses have specifically investigated the effects of artemether-lumefantrine (AL) on gametocyte carriage.

## Materials and methods

A systematic review of 62 articles published between 1998 and January 2014 was done which compares effects of AL on gametocyte carriage and malaria transmission with other ACTs and non-ACTs. AL was assessed based on its widespread usage of ACTs recommended by WHO, in the treatment for uncomplicated *P. falciparum* malaria in several African countries and the high number of clinical trials that have evaluated the product. Impact of AL on population gametocyte carriage and potential future role of AL in malaria elimination initiatives are also considered.

## Results

The gametocytocidal effect of AL was proportionately con-sis tent across the studies reviewed, despite inherent difficulties in comparing data from a range of studies that utilized different diagnostic approaches to assess baseline gametocyte counts and differences in study designs. However, the specific place of AL is the subject of ongoing research and will be dependent on its use in combination with other intervention measures, rational use of the product, interaction with other medicinal agents in situations of malaria co-infections and comorbidities, and demographic differences. Use of ACTs like AL in malaria elimination strategies will therefore require balancing potential increased roll out with rational use and protection against resistance development.

## Conclusion

AL (along with other ACTs) will continue to play a vital role in treatment of malaria by rapidly clearing asexual parasite load and reducing gametocyte carriage in both symptomatic patients and asymptomatic carriers. Consequently, AL, an ACT with this significant dual activity on asexual and sexual parasite forms, can help shrink the infectious parasite reservoir that will in turn reduce infection of mosquitoes and ultimately minimize malaria transmission.

